# IL-4Rα signalling in B cells and T cells play differential roles in acute and chronic atopic dermatitis

**DOI:** 10.1038/s41598-022-26637-6

**Published:** 2023-01-04

**Authors:** Martyna Scibiorek, Nontobeko Mthembu, Sandisiwe Mangali, Amkele Ngomti, Paul Ikwegbue, Frank Brombacher, Sabelo Hadebe

**Affiliations:** 1 Division of Immunology, Department of Pathology, Faculty of Health Sciences, University of Cape Town, Cape Town, South Africa; 2 Division of Immunology, International Centre for Genetic Engineering and Biotechnology (ICGEB), Institute of Infectious Diseases and Molecular Medicine (IDM), Health Science Faculty, University of Cape Town, Cape Town, South Africa; 3 Wellcome Centre for Infectious Diseases Research in Africa (CIDRI-Africa), Institute of Infectious Diseases and Molecular Medicine (IDM), Faculty of Health Sciences, University of Cape Town, Cape Town, 7925 South Africa

**Keywords:** Interleukins, Immunology, CD4-positive T cells, CD8-positive T cells, Gammadelta T cells, T-cell receptor

## Abstract

Atopic dermatitis (AD) is a common pruritic inflammatory skin disease with complex environmental and genetic predisposing factors. Primary skin barrier dysfunction and aberrant T helper 2 (TH2) responses to common allergens, together with increased serum IgE antibodies, characterise the disease. B and T cells are essential in the disease manifestation, however, the exact mechanism of how these cells is involved is unclear. Targeting interleukin 4 receptor alpha (IL-4Rα), an IL-4/IL-13 signalling axis, with dupilumab shows efficacy in AD. We investigated the importance of IL-4Rα signalling specifically on B and T cells during acute and chronic models of AD. We used House dust mite (HDM) and Ovalbumin (OVA) in chronic models and a low-calcemic analog of vitamin D (MC903) for acute models of AD. We used mb1^cre^IL-4Rα^−/lox^, iLCK^cre^IL-4Rα^−/lox^, LCK^cre^IL-4Rα^−/lox^, CD4^cre^IL-4Rα^−/lox^, Foxp3^cre^IL-4Rα^−/lox^ and IL-4Rα^−/lox^ littermate controls. IL-4Rα-responsive B cells were essential in serum IgE levels, but not in epidermal thickening in both chronic and acute models. IL-4Rα-responsive T cells were essential in epidermal thickening in the pan-T cell, but not CD4 or CD8 T cells suggesting the importance of γδT cells during acute AD. Our results suggest that IL-4Rα responsiveness on innate T cells regulates acute atopic dermatitis, while on B cells it regulates IgE.

## Introduction

Atopic dermatitis (AD) affects approximately 20% of children and up to 3% of adults worldwide^1^. It is a reoccurring chronic skin disorder with acute flare-ups characterized by eczematous lesions of dry skin and pruritis^2^. AD leads to a decrease in patients’ health-related quality of life and reports a higher incidence of depression, anxiety, and suicide as well as pain, fatigue, and insomnia^3–6^.

Recent studies on AD show that both intrinsic (non-allergic) and extrinsic (allergic) factors can play a role in the pathogenesis of disease progression^7^. As the endotypes vary in immunoglobulin E (IgE) levels, the type 2 cytokines namely interleukin 4 and 13 (IL-4 and IL-13) remain elevated in both types of the disease, particularly in the lesional areas of the skin^8^. Dupilumab, an interleukin 4 receptor alpha (IL-4Rα) antagonist was recently approved by FDA as a treatment for AD after it showed positive effects in patients with moderate to severe AD, with reduced blood type 2 signature, and reversed epidermal abnormalities^9^. This clinical trial provided strong evidence for IL-4/IL-13 as a key target in controlling debilitating AD disease.

IL-4Rα, a heterodimeric subunit binds to IL-4 and IL-13 for downstream signalling on hematopoietic and non-hematopoietic cells^10^. IL-4 binds to either type I receptor (IL-4Rα and γC chain shared with IL-2, IL-7, IL-9 and IL-21) or type II receptor (IL-4Rα and IL‐13Rα1)^11^. Both receptor complexes can activate phosphorylation of the signal transducer and activator of transcription (STAT6) pathway via Janus kinase^12^. Type I IL-4 receptor complex can also activate the insulin receptor substrate (IRS)-2 pathway to affect the symptoms of allergic diseases^13^. Type II receptor complex has been shown to have a much higher affinity for IL-4 compared to type I complex^12^. IL-13 is the main pathology driver and mediates its effects through the type II receptor complex (IL-4Rα and IL‐13Rα1)^14^. IL-13 is also capable of signalling independently of IL-4Rα through IL-13Rα2, which was previously thought to be a decoy receptor^15–18^.

IL-13 was found to be important in regulating epidermal thickening, whereas IL-4 was essential for systemic inflammation and anaphylactic shock, but not epidermal thickening^19^. Subsequent studies using Ovalbumin and *Aspergillus* epicutaneous allergen sensitisation and other skin irritants such as oxazolone validated the critical role of type 1 and type II IL-4Rα signalling requirement in atopic dermatitis^16^. The critical importance of IL-13 in epidermal thickening has been shown to be mediated by both IL-4Rα/IL-13Rα1 and IL-13Rα2 with the latter being more essential in keratinocyte signalling^15,16,20^. IL-4 through signalling via type I IL-4R (IL-4Rα/γC chain) is crucial for IL-4 production, IgE class switching, CCL24, and skin eosinophilia, whereas IL-13 via type II IL-4R (IL-4Rα/IL-13Rα1) is essential for epidermal hyperplasia, TNF-α, CXCL1, and CCL11 production^20^. Interestingly, IL-4Rα signalling on CD4 T cells or macrophages was shown not to be essential in epidermal hyperplasia during *Anisakis* induced AD, despite these cell types being abundant in an inflamed skin^19^. Whether the requirement of signalling of IL-4Rα in different adaptive cell types is critical in disease outcomes is unclear. Furthermore, allergens that can cause acute or chronic AD may influence the need for IL-4Rα signalling in different adaptive cells.

We investigated the possible role of IL-4Rα in different adaptive cell types using conditional knockout and acute (low-calcemic analog of vitamin D (MC903)) or chronic (House Dust Mite and Ovalbumin) allergens. We found that IL-4Rα signalling on CD4/CD8 T cells was redundant in acute and chronic AD-induced allergens, whereas IL-4Rα signalling on B cells was mainly important for regulating IgE in chronic HDM-induced AD. Interestingly, IL-4Rα signalling on γδ + T cells was essential in epidermal thickening and IgE production in calciprol-induced acute AD.

Our findings show a crucial role for IL-4Rα signalling on γδ + T cells in acute AD, but not in chronic AD models, while IL-4Rα signalling on B cells is required for IgE production.

## Results

### Chronic HDM exposure does not induce epidermal thickening but induces IgE

**Figure 1:**
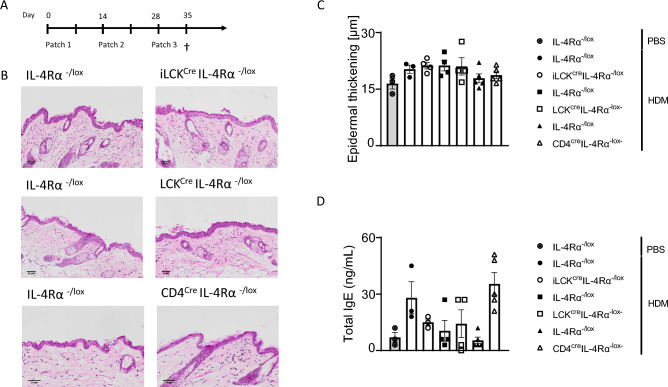
Epicutaneous sensitization with 10ug HDM induces insufficient skin inflammation. **(A)** Shaved mice (iLCK^cre^IL-4R⍺^−/lox^, CD4^cre^IL-4R⍺^−/lox^, LCK^cre^IL-4R⍺^−/lox^ and their respective IL-4R⍺^−/lox^ littermate controls) were epicutaneously sensitised via patch containing PBS or HDM 100 µg at day 0, 14 and 28 and blood and skin were collected at day 35. (**B**) Representative histology images of skin biopsies of ventral side from HDM or PBS treated mice, stained with H&E, scale bar 50 μm. (**C**) Quantification of epidermal thickening using QuPath software. (**D**) Quantification of total IgE serum by ELISA. Shown is one representative experiment with mean ± SD. Statistical analysis was performed using Student t-test Mann–Whitney where*p < 0,05,**p < 0,01 between littermate IL-4Rα^–/lox^ PBS group vs HDM challenged mice. n = 3–5 mice per group.

We have previously shown that IL-4Rα is essential in OVA and *Anisakis-*induced AD and in IL-13-mediated epidermal thickening^16,19^. Since many cell types in the skin express IL-4Rα, we set out to investigate whether different T cell subsets expressing IL-4Rα would be essential in HDM-induced AD (Fig. 1A). We observed no major differences in inguinal lymph node CD4, CD8 and B cells frequencies and numbers between iLCK^cre^IL-4Rα^−/lox^, LCK^cre^IL-4Rα^−/lox^, CD4^cre^IL-4Rα^−/lox^ mice and their respective littermate controls (Supplementary Fig. 1a,b,d,e). We observed a significant increase in γδ T cells in CD4^cre^IL-4Rα^−/lox^ and iLCK^cre^IL-4Rα^−/lox^ compared to their respective littermate controls (Supplementary Fig. 1c,e). We could also validate a decrease in IL-4Rα expression in all T cell-specific subsets compared to littermate controls except for γδ T cells in LCK^cre^IL-4Rα^−/lox^ mice as expected (Supplementary Fig. 1a–c). We compared epidermal thickening in iLCK^cre^IL-4Rα^−/lox^, LCK^cre^IL-4Rα^−/lox^, CD4^cre^IL-4Rα^−/lox^ mice to their respective littermate controls IL-4Rα^−/lox^ and IL-4Rα^−/lox^ PBS control (Fig. 1B). We did not observe any significant epidermal thickening changes between iLCK^cre^IL-4Rα^−/lox^, LCK^cre^IL-4Rα^−/lox^, CD4^cre^IL-4Rα^−/lox^ mice compared to their respective littermate control treated with HDM (Fig. 1B,C). There was no significant difference in epidermal thickening between HDM and PBS control exposed mice. Tap stripping and shaving have been shown to induce epidermal thickening independent of allergen exposure^21^. We compared epidermal thickening in naïve untreated mouse strains and found no significant differences between naïve groups (Supplementary Fig. 2a,b). To validate whether three HDM exposure was sufficient to sensitise mice we measured IgE and found increased but not significant levels in almost all HDM-exposed mice compared to PBS-exposed control (Fig. 1D). We also measured dermal mast cells and basophils by flow cytometry and found increased numbers of these cells in iLCK^cre^IL-4Rα^−/lox^ mice treated with HDM when compared to their respective littermate control or PBS treated littermate control (Supplementary Fig. 2c,d). We observed similar findings in mice deficient in IL-4Rα in T regs (Foxp3^cre^IL-4Rα^−/lox^) compared to littermate control when treated with HDM or OVA, except for increased but not significant IgE (Supplementary Fig. 3b). All together these data suggested that topical allergen exposure induces IgE but is not sufficient for epidermal thickening.

### IL-4Rα-responsiveness in all T cells mediates MC903-induced AD inflammation

**Figure 2:**
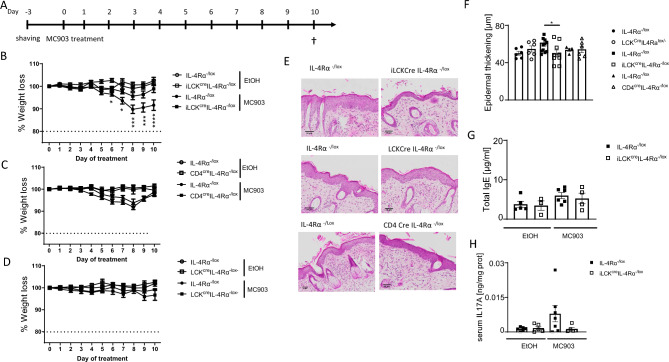
Pan T cell IL-4R⍺ responsiveness is essential in acute MC903-induced epidermal thickening. **(A)** Mice were shaved at −3 days before treatment with Ethanol or 45 µM MC903 for 9 consecutive days. (**B**) Weight loss in IL-4R⍺^−/lox^ and iLCK^cre^IL-4R⍺^−/lox^ mice. (**C**) Weight loss in IL-4R⍺^−/lox^ and CD4^cre^IL-4R⍺^−/lox^ mice. (**D**) Weight loss in IL-4R⍺^−/lox^ and LCK^cre^IL-4R⍺^−/lox^ mice. (**E**) H&E-stained images showing epidermal thickening, scale bar 50 μm. (**F**) Epidermal thickening using QuPath software, (**G**) Total IgE serum levels. (**H**) IL-17A serum levels. Shown is one representative experiment of 2 with mean ± SD. Statistical analysis was performed using Two-way ANOVA with Bonferroni post-test (**B–D**) or Student t-test Mann–Whitney (**F–H**) where *p < 0.05, **p < 0.01, ***p > 0.001, ****p > 0.0001 between IL-4Rα^–/lox^ vs iLCK^cre^IL-4Rα^–/lox^ MC903 treated mice. n = 5–8 mice per group.

To understand whether IL-4Rα would be essential in other forms of AD, such as the one induced by acute skin irritants, we compared iLCK^cre^IL-4Rα^−/lox^, LCK^cre^IL-4Rα^−/lox^, CD4^cre^IL-4Rα^−/lox^ mice to their respective littermate controls (Fig. 2A). MC903 an analogue of Vitamin D3 induces acute AD-like lesions and itch in mice when applied topically^22^. We optimised the concentration of MC903 to apply in our setting and set 45 μM as sufficient and stable to induce AD-like symptoms (Supplementary Fig. 4a). We observed no significant differences in weight loss and epidermal thickness between the vehicle (EtOH) treated iLCK^cre^IL-4Rα^−/lox^, LCK^cre^IL-4Rα^−/lox^, CD4^cre^IL-4Rα^−/lox^ mice and their respective littermate controls (Fig. 2B–D, Supplementary Fig. 4b). We also observed no significant differences in weight loss (Fig. 2C,D) and epidermal thickness (Fig. 2E,F) between LCK^cre^IL-4Rα^−/lox^, CD4^cre^IL-4Rα^−/lox^ mice compared to their respective littermate controls when treated with 45 μM MC903. Interestingly, we observed a significant decrease in weight loss (Fig. 2B) starting at day 5 of treatment in IL-4Rα^−/lox^ littermate control mice compared to iLCK^cre^IL-4Rα^−/lox^ which was accompanied by striking epidermal thickening (Fig. 2E,F). Total IgE levels in serum were similar in iLCK^cre^IL-4Rα^−/lox^ mice treated with MC903 when compared to the littermate control treated with the same substance (Fig. 2G). Cytokine, IL-17A which is upregulated during MC903 induced AD was also reduced slightly in iLCK^cre^IL-4Rα^−/lox^ compared to littermate control (Fig. 2H). Type 2 cytokine, IL-4 in serum was significantly changed between iLCK^cre^IL-4Rα^−/lox^ compared to littermate control but not IL-13 (Supplementary Fig. 5a) These data suggested that the lack of IL-4Rα in all T cells protects against acute MC903-induced AD.

### IL-4Rα-responsiveness in all T cells mediates cytokine production in acute AD

**Figure 3:**
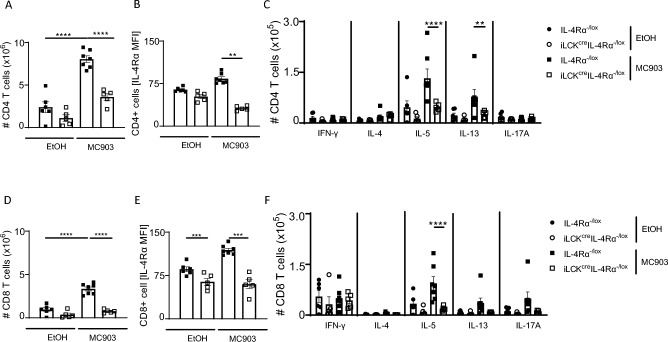
Pan T cell IL-4R⍺ responsiveness is essential in acute MC903-induced atopic dermatitis. (**A**) CD4 T cell numbers in inguinal lymph nodes in IL-4R⍺^−/lox^ littermate and iLCK^cre^IL-4R⍺^−/lox^ treated with EtOH and MC903. (**B**) IL-4R⍺ expression in CD4 T cells. (**C**) CD4 T cell cytokine production (IFN-γ, IL-4, IL-5, IL-13 and IL-17A). (**D**) CD8 T cells numbers in iLNs. (**E**) IL-4R⍺ expression in CD8 T cells. (**F**) CD8 T cell cytokine production (IFN-g, IL-4, IL-5, IL-13 and IL-17A). Shown are 2 pooled experiments with mean ± SEM. Statistical analysis was performed using the Mann–Whitney Student t-test where *p < 0,05, **p < 0,01, ***p < 0.001, ****p < 0.0001 between knockout and its respective littermate IL-4Rα^–/lox^ control in either EtOH group or MC903 treated mice. n = 5–8 mice per group.

We then measured CD4 and CD8 T cells in iLN between iLCK^cre^IL-4Rα^−/lox^ and littermate control treated with vehicle or MC903. Although the number of CD4 T cells was increased in mice treated with MC903 compared to vehicle-treated mice, there was a significant difference between iLCK^cre^IL-4Rα^−/lox^ compared to the littermate control (Fig. 3A). This also translated to a significant reduction in IL-4Rα expression in iLCK^cre^IL-4Rα^−/lox^ compared to littermate control (Fig. 3B). We checked for intracellular production of cytokines by these CD4 T cells and found a significant reduction in CD4 T cells producing IL-5 and IL-13, but not IFN-γ or IL-17A in iLCK^cre^IL-4Rα^−/lox^ compared to littermate control (Fig. 3C). We observed similar reductions in iLN CD8 T cells (Fig. 3D), IL-4Rα expression (Fig. 3E) and CD8 T cells production producing IL-5, IL-13 and IL-17A in iLCK^cre^IL-4Rα^−/lox^ compared to littermate control (Fig. 3F). We observed higher numbers and frequencies of γδ T cells in MC903-treated and vehicle-treated iLCK^cre^IL-4Rα^−/lox^ mice compared to littermate controls, like our observations in HDM-treated mice (Data not shown). Overall, these data pointed to a redundant role of IL-4Rα signalling in CD4 and CD8 T cells, but a requirement of this receptor in γδT cells as mice deficient of this receptor in all T cells were protected from acute AD.

### IL-4Rα responsive B cells are not essential in chronic HDM AD but regulate IgE production

**Figure 4:**
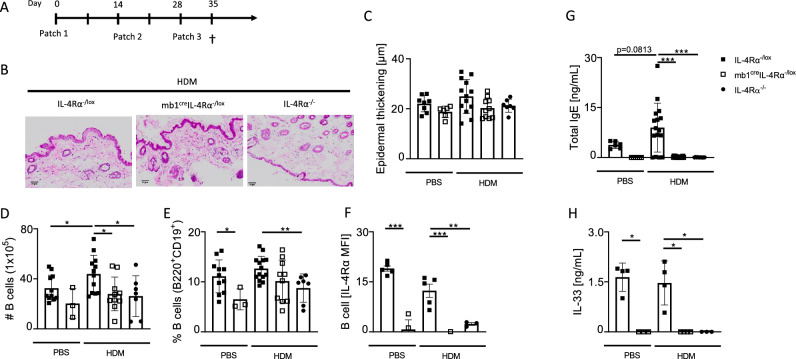
Epicutaneous sensitization with HDM induces insufficient skin inflammation, but IgE-dependent IL-4R⍺ expression in B cells. Shaved mice (mb1^cre^IL-4R⍺^−/lox^, IL-4R⍺^−/lox^ littermate controls and IL-4R⍺^−/−^) were epicutaneously sensitised as in Fig. 1. (**A**) H&E-stained images showing epidermal thickening, scale bar 50 μm. (**B**) Epidermal thickening using QuPath software. (**C**) Total B cell numbers in the iLNs. (**D**) Frequency of B cells (Live + singlets, lymphocytes, CD19^+^BB20^+^MHCII^+^) in the iLNs. (**E**) IL-4R⍺ expression in B cells. (**F**) Total IgE serum levels. (**G**) IL-33 serum levels. Shown are 2 pooled experiments with mean ± SEM. Statistical analysis was performed using Student t-test Mann–Whitney where*p < 0,05, **p < 0,01, ***p < 0.001 between littermate IL-4Rα^–/lox^ vs mb1^cre^IL-4R⍺^−/lox^ or IL-4R⍺^−/−^ mice. n = 3–12 mice per group.

B cells secreting IgE have been shown to be essential in tumour surveillance in the skin^21,23^. These B cells were shown to receive their IL-4 signal via γδT cells allowing IgE class switching by B cells^23^. Given that we had shown indirectly a role for IL-4Rα in γδT cells in acute MC903-induce AD, we wondered whether IL-4Rα responsiveness by B cells would be important in both chronic and acute models of AD. We epicutaneously sensitised mb1^cre^IL-4Rα^−/lox^, IL-4Rα^−/-^ and littermate controls IL-4Rα^−/lox^ mice to HDM with IL-4Rα^−/lox^ mice exposed to PBS serving as controls (Fig. 4A). We found reduced epidermal thickening in mb1^cre^IL-4Rα^−/lox^ and IL-4Rα^−/-^ mice sensitised to HDM when compared to littermate controls sensitised to HDM although this did not reach significance (Fig. 4B,C). We then measured cellular infiltrate in the iLNs and found significantly higher total B cells and frequencies in littermate control mice sensitised to HDM when compared to mb1^cre^IL-4Rα^−/lox^, IL-4Rα^−/-^ mice and PBS control mice (Fig. 4D,E). To validate the deletion of IL-4Rα on B cells, we showed a significant reduction in IL-4Rα in mb1^cre^IL-4Rα^−/lox^ mice compared to littermate controls and no expression of IL-4Rα in IL-4Rα^−/−^ mice as expected (Fig. 4F). We then measured total IgE in serum and found significantly increased IgE in littermate controls sensitised to HDM when compared to mb1^cre^IL-4Rα^−/lox^ and IL-4Rα^−/-^ sensitised to HDM (Fig. 4G). We observed increased but not significantly changed IL-33 secretion in both PBS and HDM sensitised littermate control mice, which was absent in both mb1^cre^IL-4Rα^−/lox^ and IL-4Rα^−/−^ mice (Fig. 4H). This suggested an IL-4Rα-dependent and allergen-independent IL-33 secretion. Interestingly, in the skin, we did not observe any changes in mRNA expression of il-33 or any other type 2 or type 17 transcripts (Supplementary Fig. 6a).

### IL-4Rα responsive B cells are not essential in acute AD-induced skin inflammation but regulate IgE production

**Figure 5:**
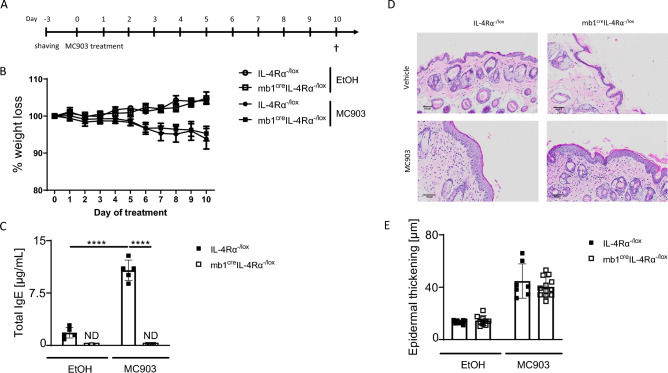
IL-4R⍺ responsiveness in B cells is not essential in acute MC903-induced epidermal thickening but is required for IgE production. **(A)** Mice were shaved 3 days prior to topical treatment with either Ethanol or 45 µM MC903 for 9 consecutive days. (**B**) Weight loss in IL-4R⍺^−/lox^ and mb1^cre^IL-4R⍺^−/lox^ treated with EtOH and MC903. (**C**) Total IgE serum levels. (**D**) H&E-stained images showing epidermal thickening, scale bar 50 μm. (**E**) Quantification of epidermal thickening using QuPath software, Shown are pooled 2 independent experiments with mean ± SEM. Statistical analysis was performed using the Student t-test Mann–Whitney where ****p > 0.0001 between littermate IL-4Rα^–/lox^ group vs iLCK^cre^IL-4Rα^–/lox^ group treated with EtOH or MC903. n = 5–10 mice per group.

To further understand the dynamics of IL-4Rα responsive B cells in acute AD, we treated mb1^cre^IL-4Rα^−/lox^ and IL-4Rα^−/lox^ littermate controls with a skin irritant MC903 (Fig. 5A). We monitored weight loss over 10 days and found MC903 mice to lose weight at similar levels starting at day 5. Mice treated with vehicle (EtOH) did not lose weight during this time (Fig. 5B). We measured total serum IgE and found significantly increased IgE in littermate control mice exposed to MC903 compared to mb1^cre^IL-4Rα^−/lox^ mice or mice lacking IL-4Rα in all cells or mice deficient in B cells (Fig. 5C). We then measured epidermal thickening on day 10 and found increased but not significantly different thickness between mb1^cre^IL-4Rα^−/lox^ and littermate control treated with MC903 (Fig. 5D,E). Similar results were observed in vehicle-treated mice except for low levels of epidermal thickening was observed (Fig. 5D,E). There was a decreased but not significant type 2 and type 17 mRNA expression in the skin between mb1^cre^IL-4Rα^−/lox^ and littermate control treated with MC903 (Supplementary Fig. 6b). Overall, these results suggested that IgE production was dependent on IL-4Rα expressing B cells.

### IL-4Rα responsive B cells are required for germinal centre (GC) formation and class switching in acute AD-induced skin inflammation

**Figure 6:**
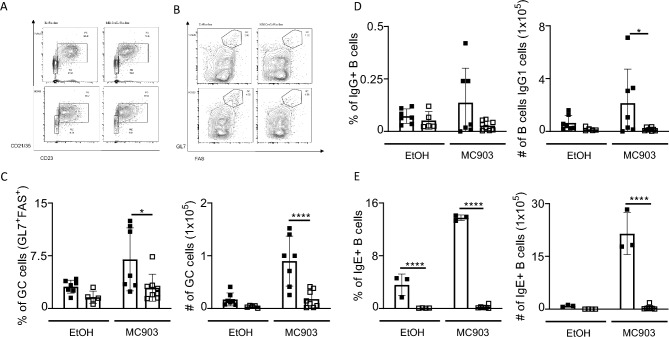
IL-4R⍺ responsiveness in B cells essential in GC and class switching during acute MC903-induced atopic dermatitis. **(A)** Representative FACS plots of Follicular (FO) and marginal zone (MZ) B cells in inguinal lymph nodes between IL-4R⍺^−/lox^ and mb1^cre^IL-4R⍺^−/lox^ treated with EtOH and MC903. (**B**) Representative FACS plots of Germinal Centre (GCs) B cells in inguinal lymph nodes. (**C**) Frequency and the total number of GC B cells. (**D**) Frequency and the total number of IgG1 + B cells. (**E**) Frequency and the total number of IgE + B cells. Shown are 2 pooled experiments with mean ± SEM. Statistical analysis was performed using Student t-test Mann–Whitney where *p < 0,05, **p < 0,01 between littermate IL-4Rα^–/lox^ EtOH group vs MC903 treated mice. n = 5–8 mice per group.

We further investigated the importance of IL-4Rα signalling on B cells in class switching during skin irritant-induced acute AD. We measured the frequencies of B cell populations in the iLN. We observed no significant changes in follicular (FO) or marginal zone (MZ) B cells between mb1^cre^IL-4Rα^−/lox^ mice and littermate control mice in both vehicles and MC903 treated mice (Fig. 6A). We measured frequencies and number of GCs (FAS^+^GL7^+^) in iLN and found significantly reduced GCs frequencies and numbers in mb1^cre^IL-4Rα^−/lox^ mice compared to littermate controls exposed to MC903 (Fig. 6B,C). The frequency and number of IgG1 and IgE expressing B cells were also significantly reduced in mb1^cre^IL-4Rα^−/lox^ mice compared to littermate controls (Fig. 6D,E), which corroborated our earlier findings of reduced total IgE in these mice.

## Discussion

The importance of TH2-targeted therapeutics has been vastly studied in the context of atopic dermatitis^20,24–29^. The recently recommended treatment is systemic administration of monoclonal antibody targeting IL-4Rα—dupilumab, in conjunction with topical glucocorticosteroids^30^. Such a regime is highly effective in alleviating AD clinical symptoms, and a reduction in IL-22 or eotaxin, as well as total IgE. However, clinical studies show an increase in IL-4 and IL-13 upon the treatment as a resultant side effect^31,32^. The most often reported side effect is conjunctivitis and blood eosinophilia. These studies suggest that despite high efficacy, the treatment could still be improved. Here, we used acute and chronic models of AD to understand the cell-specific requirements of IL-4Rα in adaptive cells, mainly CD4, CD8 and B cells. We found that IL-4Rα expressed by innate γδT cells was essential in MC903-induced acute AD, whereas IL-4Rα expressed by all T cells were redundant in chronic HDM or OVA-induced AD. We also found that IL-4Rα expressed by B cells was essential in chronic HDM-induced AD and regulated IgE.

Previously our group showed that the reduction of IL-4Rα expression on LCK^cre^ IL-4Rα^−/lox^ mouse strain contributes to the reduction of IL-5 and IL-4 but not IL-13 in the *Anisakis*-induced model of AD^19^. We further showed that in absence of IL-13 but not IL-4, we can reduce the effect of Anisakis application like epidermal hyperplasia and cellular infiltration. Bitton et al. described a mechanism of protection from oxazolone-induced AD murine model by inhibition of IL-13 detection via IL-13Rα1^20^. Our group has also shown an IL-4Rα-independent function of IL-13 in OVA-induced AD^16^. Here, we showed that IL-4Rα responsiveness in T cells was redundant in chronic HDM models as mice lacking IL-4Rα in CD4 alone, CD4 and CD8 or CD4, CD8 and γδ T cells. We also did not observe any changes in IgE levels. Previous studies have shown that the lack of GATA3 in Foxp3 T regs led to the poor accumulation of T regs in lymphoid tissues and autoimmunity^33^. T regs have been shown to be essential in skin homeostasis where they are crucial in promoting hair follicle stem cell regeneration^34^. In our study, deficiency of IL-4Rα in Foxp3 T reg cells did not lead to adverse skin inflammation in chronic AD models but led to an increase in total IgE. These findings suggest that although local skin inflammation is not impacted by a deficiency in IL-4Rα in T reg cells, it may have an impact on systemic IgE dysregulation. It is possible that HDM and OVA allergens were not sufficient to induce a keratinocyte mechanical injury required for skin sensitisation as previously observed^35–38^.

In acute AD models, we found that mice lacking IL-4Rα in pan T cells were protected from MC903, shown by a transient loss in weight, reduced epidermal thickening and reduced type 2 cytokine production by CD4 and CD8 T cells. This contrasted with mice lacking IL-4Rα only in CD4 and CD8 T cells, which were susceptible to similar levels to littermate controls. This suggested that the IL-4Rα protective effect observed in pan T cell IL-4Rα-deficient mice might be γδ T cell-derived, and these cells could potentially influence B cell function.

B cells can be both IL-4-responsive due to the presence of the IL-4 receptor on the cell surface, as well as secrete the cytokine, so the deletion of the receptor can have both autocrine and paracrine consequences^39^. The effect of IL-4/IL-13 binding on IL-4Rα is required for B cell maturation, formation of GCs, B-T cell interaction and adequate antibody class switched IgE production^40,41^. Interestingly, in the skin γδ T cells have been shown to be essential in producing IL-4 production and influencing B cell class switching to IgE, which is essential in skin tumour surveillance^23,42^. We found that mice deficient in IL-4Rα on B cells had little impact on epidermal skin thickening but were required in IgE production, Tfh cells and GC formation. This is consistent with our previous studies in allergic asthma where the absence of IL-4Rα on B cells led to reduced GC numbers, Tfh and IgE production^43^. Despite IgE being essential in skin prick testing and in mast cell and basophil activation, its role in epidermal hypertrophy is limited^44^. Mice deficient of IgE can develop epidermal hyperplasia when sensitised to OVA^44^. Our data is consistent with these findings where we show that IgE production dependent on IL-4Rα responsive B cells is redundant in epidermal hyperplasia. Cell-specific factors that influence AD are not known and IL-4Rα, a key factor in AD, is expressed by many cell types. IL-4Rα expressed by γδ T cells is essential in the pathogenesis of acute AD, while IL-4Rα expressed by other T cells is not important. IL-4Rα expressed by B cells is important in IgE production in both acute and chronic AD.

Overall, we showed mild allergic sensitization due to the lack of induction of epithelial damage by mechanical tape-stripping in OVA and HDM models. Other studies on AD have shown various combinations of HDM strain *Dermatophagoides farina* to be efficient in the induction of AD-like lesions when boosted with skin irritants such as MC903, or capsaicin or with bacterial toxins like *Staphylococcal enterotoxin* B from *S. aureus*^45–47^. We were confident that our epicutaneous sensitisation model with HDM primed skin resident cells, as we were able to observe atopic march characterised by increased allergic airway inflammation which was dependent on IL-4Rα expression by B cells (Data not shown).

In human AD, IL-4/IL-13 signalling is central in the pathogenesis of the disease, with increased colonisation of the skin with *S. aureus*^48^, where both cytokines favour *S. aureus* adhesion and keratinocyte killing^48^. At baseline, in skin lesion areas, there is less microbial diversity with a dominant *S aureus*. After 16-week treatment with dupilumab, *S. aureus* is decreased in skin lesion areas and the diversity of other microbial skin commensals is increased^9,48^. In human clinical trials, it is unclear whether there is an increased abundance of commensal microbiota that could be limiting *S. aureus*. The long-term implications of reducing pro-tissue repair mechanisms by cytokines IL-4 and IL-13 are currently unknown. Early *S. epidermis* skin colonisation has a long-term effect on T-cell priming, non-classical MHC I activation and the activation of mucosal-associated invariant T-cells^49^.

## Conclusions

In this article, we show that therapeutic targeting of IL-4Rα expressing cells, particularly those of adaptive immunity may need clarification based on allergen and chronicity of the disease where acute cases characterised by itch may benefit from targeting innate T cells and chronic cases may benefit from targeting B cells and IgE secretion. Personalised therapeutics aimed at TH2 diseases require a clear understanding of the role of each cell type.

## Material and methods

### Ethical declarations for animal experiments

Animal procedures were performed according to strict recommendations by the South African Veterinary Council and were approved by the University of Cape Town Animal Ethics Committee (Reference number 017/004, 021/006). All authors complied with the ARRIVE guidelines and institutional guidelines on the use of animals in research.

### Mice

All mice used in this study were generated at the University of Cape Town Animal Research Facility. Original cre strains of mice were purchased from Jackson Laboratories and IL-4Rα^lox/lox^ was generated in house^50^. To generate mice deficient of IL-4Rα only on B cells (mb1^cre^IL-4Rα^−/lox^), CD4 T cells only (CD4^cre^IL-4Rα^−/lox^), CD4/CD8 T cells only (LCK^cre^IL-4Rα^−/lox^), T regs (Foxp3^cre^IL-4Rα^−/lox^) or pan-T cells (iLCK^cre^IL-4Rα^−/lox^) we intercrossed homozygous mb1^cre^ mice^51^ or CD4^cre^ mice^52^ or LCK^cre^ mice^53^ or iLCK^cre^ mice or Foxp3^cre^ mice^54^ with homozygous IL-4Rα^lox/lox^ mice^50^ to generate hemizygous mb1^cre^IL-4Rα^−/lox^^55^, CD4^cre^IL-4Rα^−/lox^^53^, LCK^cre^IL-4Rα^−/lox^^53^, iLCK^cre^IL-4Rα^−/lox^ or Foxp3^cre^IL-4Rα^−/lox^^54,56^. Hemizygous littermates (IL-4Rα^−/lox^) expressing a single functional IL-4Rα allele was used as a wild-type control in all experiments. In some instances, mice lacking B cell (μMT^−/-^)^57^ in Balb/c background were used as controls. Mice were housed in independently ventilated cages under specific pathogen-free conditions at the University of Cape Town Animal Facility. All mice were used at eight to 10 weeks of age.

### Chronic atopic dermatitis models

Mice were anesthetized with ketamine (Anaket-V; Centaur Labs, Johannesburg, South Africa) and xylazine (Rompun; Bayer, Isando, South Africa) mix and shaved on ventral side 1 × 1 cm with single-blade disposable razor (BIC, South Africa). Mice were treated with 100 μg of House Dust Mite (Stellergens Greer Laboratories, Lenoir, USA) or 200 μg ovalbumin (OVA, grade V; Sigma-Aldrich, South Africa) diluted in 200 µL of Phosphate-Buffered Saline (PBS, Thermofisher, South Africa) or with PBS as control on a sterile band aid (Elastoplast, Baiersdorf, South Africa) patch which was removed after 24 h. Treatments were done on day 0, 14 and 28 and mice were killed on day 35.

### Acute atopic dermatitis model

Mice were shaved using a single-blade disposable razor (BIC, South Africa) on the ventral side 1 × 1 cm, 3 days prior to the start of the experiment. Mice were treated with 100 µL of 45uM calcineurin inhibitor, MC903 (Tocris Bioscience, United Kingdom) in 100% ethanol (EtOH, Thermofisher, South Africa) or with 100% EtOH as a control vehicle for 10 consecutive days. Mice were weighed daily to monitor weight loss and general welfare. Mice were killed on day 8 or 11 and organs were collected for further analysis.

### Sample collection and processing of skin tissue

Mice were euthanized using by halothane (Piramal Healthcare Limited, India) inhalation, blood collected via cardiac puncture and ventral side of the skin shaved. Patch size of the skin (19 mm × 10 mm) was cut out and placed in 4% formalin for histology analysis. Inguinal lymph nodes were collected into non-supplemented RPMI-1640 Medium (Thermofisher, South Africa) and processed as single-cell suspensions before counting in trypan blue.

### Flow cytometry

Inguinal lymph node single cells were seeded 2 × 10^6^ cells per well in 96-well U-bottom shaped plate (Thermo Scientific, USA) and were stained with surface cell markers in FACS buffer (1% BSA, 2% inactivated rat serum, 1% Fc block). Antibodies used in these experiments included, phycoerythrobilin (PE)- conjugated anti-Siglec-F (clone, E50-2440), anti-CD124 (IL-4Rα, clone, M-1), anti-CD44 (clone, KM114), FITC- conjugated anti-Gr-1 (clone, RB6-8C5), CD45 (clone, 30-F11), PerCP Cy5.5- conjugated anti-Ly6C (clone, AL-21), -CD45.1 (clone, A20), Allophycocyanin (APC)- conjugated anti-CD11c (clone, HL3), anti-FoxP3 (clone, MF23), V450 conjugated anti-CD11b (clone, M1/70), anti-CD62L (clone, MEL-14), anti-IgG1 (clone, A110-1), AlexaFlour 700- conjugated anti-CD3ε (clone, 145-2C11), V500- anti-CD4 (clone, RM4-5) and anti- B220 (clone, RA3-6B2), APC-Cy7-conjugated anti-CD19 (clone, 1D3) and anti-CD8 (clone, 53-6.7), BV786 conjugated anti-IgE (clone, R35-72) and anti-IL-33R (ST2) (clone, U29-93), biotin-CD25 (clone, 7D4) were purchased from BD Pharmingen (San Diego, CA). PE-Cynanine7 anti-F4/80 (clone, BM8), AlexaFlouro 700- conjugated anti-MHC II (clone, M5/114) and Live/dead Fixable Yellow stain (Qdot605 dead cell exclusion dye) were purchased from eBiosciences. Biotin-labelled antibodies were detected by Texas Red conjugated PE (BD Biosciences).

### Intracellular staining

For intracellular cytokine staining, cells were restimulated with phorbal myristate acetate (Sigma-Aldrich) (50 ng/mL), ionomycin (Sigma-Aldrich) (250 ng/mL), and monensin (Sigma-Aldrich) (200 mM in IMDM/10% FCS) for 5 h at 37˚C then fixed in 2% PFA, permeabilised with Foxp3 transcriptional factor staining buffer kit (eBioscience) before intracellular staining with appropriate cytokine antibodies, FITC anti IL-4 (clone, 11B11), PE anti-IL-5 (clone, TRFK5), PE-Cy7 anti-IL-13 (clone, eBio13A), PerCP Cy5.5 anti-IL-17A (clone, TC11-18H10), and acquisition through LSR Fortessa machine (BD Immunocytometry system, San Jose, CA, USA) and data was analysed using Flowjo software (Treestar, Ashland, OR, USA).

### Histology

Formalin-fixed (4%) skin biopsies from mice were stained with haematoxylin and eosin (H&E). Image acquisition was done using VS120 Virtual Slide Microscope (Olympus Global, USA) 20× magnification and quantification of epidermal thickening was performed using QuPath.Ink open software.

### Antibody ELISAs

For antibody ELISAs, the plate was coated with using 5 μg/ml HDM for specific IgGs. Total IgE in serum was measured using anti-mouse IgE (BD Biosciences, 553413) to coat, mouse IgE (κ, anti-TNP, BD Biosciences, 557079) as standard and biotin anti-mouse IgE (BD Biosciences, 553419) as secondary antibody.

### In vitro cytokine ELISAs

For in vitro cytokine production analysis, single cell suspensions were prepared from inguinal lymph nodes of treated and control mice. Cells (2 × 10^5^ cells, in 200µL) were incubated for 5 days in IMDM/10% FCS (Delta Bioproducts, Kempton Park, South Africa) in 96-well plates. Cells were either stimulated with HDM (30 µg/mL), OVA (30 µg/mL) or anti-CD3 (10 µg/mL) and supernatants were collected after a 5-day incubation period. Concentrations of IL-4, IL-5 (BD Biosciences) and IL-13 (R&D Systems, Minneapolis, Minn), were measured using ELISA assays according to the manufacturer’s protocol.

### Statistical analysis

P-values were calculated in GraphPad Prism 6 (GraphPad Software, Inc) by using a nonparametric Mann–Whitney Student's t-test or Two-way ANOVA with Bonferroni's post-test for multiple comparisons, and results are presented as the standard error of the mean (SEM) or mean of standard deviation (SD). Differences were considered significant if P was < 0.05.

## Supplementary Information

Supplementary Information.

## Data Availability

All data generated or analysed during this study are included in this published article.
