# Author Correction: IL-4Rα signalling in B cells and T cells play differential roles in acute and chronic atopic dermatitis

**DOI:** 10.1038/s41598-023-36670-8

**Published:** 2023-06-14

**Authors:** Martyna Scibiorek, Nontobeko Mthembu, Sandisiwe Mangali, Amkele Ngomti, Paul Ikwegbue, Frank Brombacher, Sabelo Hadebe

**Affiliations:** 1 Division of Immunology, Department of Pathology, Faculty of Health Sciences, University of Cape Town, Cape Town, South Africa; 2 Division of Immunology, International Centre for Genetic Engineering and Biotechnology (ICGEB), Institute of Infectious Diseases and Molecular Medicine (IDM), Health Science Faculty, University of Cape Town, Cape Town, South Africa; 3 Wellcome Centre for Infectious Diseases Research in Africa (CIDRI‑Africa), Institute of Infectious Diseases and Molecular Medicine (IDM), Faculty of Health Sciences, University of Cape Town, Cape Town, 7925 South Africa

Correction to: *Scientific Reports* 10.1038/s41598-022-26637-6, published online 04 January 2023

The Funding section in the original version of this Article was incomplete.

“This work was supported by ICGEB, Cape Town Component, Medical Research Council (MRC) South Africa as well as support by the South African National Research Foundation (NRF) Research Chair initiative (SARChi) and Wellcome Trust CIDRI-Africa (203135Z/16/Z) to FB. SH is supported by NRF Thuthuka Grant (117721), NRF Competitive Support for Unrated Researcher (138072), MRC South Africa under a Self-initiated grant. MS was supported by ICGEB Arturo Falaschi PhD Fellowship NM was supported by South African MRC PhD Fellowship. AN was supported by NRF MSc Scholarship.”

now reads:

“This work was supported by ICGEB, Cape Town Component, Medical Research Council (MRC) South Africa as well as support by the South African National Research Foundation (NRF) Research Chair initiative (SARChi) and Wellcome Trust CIDRI-Africa (203135Z/16/Z) to FB. SH is supported by NRF Thuthuka Grant (117721), NRF Competitive Support for Unrated Researcher (138072), MRC South Africa under a Self-initiated grant. MS was supported by ICGEB Arturo Falaschi PhD Fellowship NM was supported by South African MRC PhD Fellowship. AN was supported by NRF MSc Scholarship. This research was funded in whole, or in part, by the Wellcome Trust [Grant number 203135Z/16/Z]. For the purpose of open access, the author has applied a CC BY public copyright licence to any Author Accepted Manuscript version arising from this submission.”

The original Article has been corrected.

