# Supplementary material for: IL-4Rα signalling in B cells and T cells play differential roles in acute and chronic atopic dermatitis
Source: Sci Rep. 2023 Jan 4;13:144. doi: 10.1038/s41598-022-26637-6 (PMC9812985; doi:10.1038/s41598-022-26637-6)
Supplement: Supplementary file 1 — Supplementary Information. [file 41598_2022_26637_MOESM1_ESM.docx]

**Supplementary Figures**

IL-4Rα signalling in B cells and T cells play differential roles in acute and chronic atopic dermatitis

Martyna Scibiorek^a,b*^, Nontobeko Mthembu^a*^, Sandisiwe Mangali^a,b^, Amkele Ngomti^a,^ Paul Ikwegbue^a^, Frank Brombacher^a,b,c*^ Sabelo Hadebe^a*^,

**Supplementary Figure 1. Epicutaneous sensitization to HDM increases γδ T cells in mice deficient in IL-4R⍺ in T cells.**

1. Frequency, total number of CD4 T cells (Live^+^FSC-A^+^Singlets, Lymphocytes, CD3^+^CD4^+^) in iLN and IL-4R⍺ expression depicted as MFI in iLCK^cre^IL-4R⍺^-/lox^, CD4^cre^IL-4R⍺^-/lox^, LCK^cre^IL-4R⍺^-/lox^ and their respective IL-4R⍺^-/lox^ littermate controls treated with HDM.
2. Frequency, total number of CD8 T cells (Live^+^FSC-A^+^Singlets, Lymphocytes, CD3^+^CD8^+^) in iLN and IL-4R⍺ expression depicted as MFI in iLCK^cre^IL-4R⍺^-/lox^, CD4^cre^IL-4R⍺^-/lox^, LCK^cre^IL-4R⍺^-/lox^ and their respective IL-4R⍺^-/lox^ littermate controls treated with HDM.
3. Frequency, total number of gamma delta T cells (Live^+^FSC-A^+^Singlets, Lymphocytes, CD3^+^γδTCR^+^) in iLN and IL-4R⍺ expression depicted as MFI in iLCK^cre^IL-4R⍺^-/lox^, CD4^cre^IL-4R⍺^-/lox^, LCK^cre^IL-4R⍺^-/lox^ and their respective IL-4R⍺^-/lox^ littermate controls treated with HDM.
4. Frequency, total number of B cells (Live+FSC-A+Singlets, Lymphocytes, CD3-B220+)in iLN and IL-4R⍺ expression depicted as MFI in iLCK^cre^IL-4R⍺^-/lox^, CD4^cre^IL-4R⍺^-/lox^, LCK^cre^IL-4R⍺^-/lox^ and their respective IL-4R⍺^-/lox^ littermate controls treated with HDM.
5. Gating strategies for FACS analysis of T cell subpopulations. MFI for IL-4R⍺ was determined from each population.

Shown is one representative experiment of 2 with mean ± SD. Statistical analysis was performed using Mann-Whitney Student t-test where*p<0,05, **p<0,01, ****p<0.0001 between knockout and its respective littermate IL-4Rα^–/lox^ control in HDM treated mice. n=5-7 mice per group.

**Supp Figure 2. Epicutaneous sensitization to HDM increases dermal mast cells and basophils in mice deficient of IL-4R⍺ in T cells, while no changes are observed in epidermal thickening of naive mice between T cell IL-4R⍺ knockout groups.**

A) Comparison of histology images of skin biopsies of ventral side from naive mice stained with H&E, scale bar 50um.

B) Quantification of epidermal thickening using QuPath software,

C) Dermal mast cells (Live^+^, FCS-A, Lineage^-^, CD11b^-^, FcεR1^+^, c-Kit^low^),

D) Dermal Basophils (Live^+^, FCS-A, Lineage^-^, CD11b^+^, FcεR1^+^, c-Kit^+^), were isolated by digesting skin and separating epidermal and dermal layers and stained antibodies and numbers enumerated by Flow cytometry.

Shown is one experiment representing n=3-4 mice per group (C-D) and one representative experiment of two independent experiments, n = 3-6 mice per group.

**Supplementary Figure 3. IL-4Rα expression in T reg cells is not essential for epidermal thickening induced by HDM or Ovalbumin but may regulate IgE production.**

1. Shaved mice (Foxp3^cre^IL-4R⍺^-/lox^ and IL-4R⍺^-/lox^ littermate controls) were treated as in Figure 1. H&E stained images showing epidermal thickening, scale bar 50um.
2. Quantification of histopathology using QuPath software.
3. Total IgE levels in HDM treated mice.
4. Total IgE levels in OVA treated mice.

One representative experiment of two experiments is shown with mean ± SD. Statistical analysis was performed to define differences between knockout and littermate IL-4Rα^–/lox^ control in HDM or PBS-treated mice. n=4-6 mice per group.

**Supplementary Figure 4 The epicutaneous treatment with vehicle (EtOH) does not induce strong inflammatory response.**

1. Representative images of Ethanol and increasing doses of MC903 at day 6 of treatment.
2. Representative images of skin biopsies from ventral side, histopathology stained with H&E showing epidermal thickening, scale bar 50um.

One representative experiment, shown is mean ± SD. Statistical analysis was performed using Mann-Whitney Student t-test where*p<0,01, defining differences between knockout and its respective littermate IL-4Rα^–/lox^ control in PBS treated mice. n=3-5 mice per group.

**Supplementary Figure 5. Systematic IL-4 is changed but not IL-13 in IL-4R⍺ responsive Pan T cells in acute MC903 induced atopic dermatitis.**

IL-13 and IL-4 serum levels in IL-4R⍺^-/lox^ littermate and iLCK^cre^IL-4R⍺^-/lox^ treated with MC903 measured by ELISA.

One representative experiment, shown is mean ± SD. Statistical analysis was performed using Mann-Whitney Student t-test where **p<0,01, defining differences between knockout and its respective littermate IL-4Rα^–/lox^ control mice. n=5-6 mice per group.

**Supplementary Figure 6. No major changes in skin mRNA expression of type 2 and type 17 cytokines in B cell responsive IL-4R⍺ mice during acute and chronić AD-models.**

1. Relative expression of il-4, il-5, il-13, il-33 and il-17 in HDM epicutenously sensitised mice between littermate controls and mb1^cre^IL-4R⍺^-/lox^ mice.
2. Relative expression of il-4, il-5, il-13, il-33 and il-17 in acute MC903 induced atopic dermatitis between littermate controls and mb1^cre^IL-4R⍺^-/lox^ mice.

Shown in one experiment per model SD + Mean. n= 3-6 mice per group.

**Additional Supplementary methods**

**Skin processing**

The shaved skin from mouse was collected as described previously. The processing protocol was adopted from Jensen et al. with modifications^1^. The skin was sterilized by emersion for 1 min. before further processing in the following order: 10% BETADINE Antiseptic Solution (10% povidone-iodine), 70% ethanol (Thermofisher, South Africa), 1x sterile PBS (Thermofisher, South Africa). The tissue was soaked in 32U/mL trypsin (Gibco, USA) with epidermal side up for 3 hrs at 37 ^0^C. Consecutively, the epidermis was scrapped off using sterile surgical blade. The dermis was further transferred to sterile Hank’s balanced salt solution (HBSS, MERCK, USA) for subsequent secondary digestion. The epidermis was sieved through 70 µm cell strainer into 50 mL Falcon tubes with Roswell Park Memorial Institute -1640 (RPMI-1640) medium (Thermofisher, South Africa). The epidermis solution was further spun at 800x g for 10 min at room temperature. The supernatant was discarded and cell pellet was re-suspended for cell count and staining using 1mL RPMI-1640 medium (Thermofisher, South Africa). The dermis was minced using sterile scissors and re-suspended in 5 mL of HBSS solution supplemented with 3 mM Calcium chloride (Sigma, USA) containing 150 U/mL collagenase type IV from *Clostridium histolyticum* (Sigma, USA) in 15 mL Falcon tubes. The secondary digestion was done in incubator at 220 rpmi at 37 ^0^C for 1 hr. The single-cell suspension was sieved via 70 µm cell strainer into 50 mL Falcon tubes and re-suspended with 15 mL RPMI-1640 medium (Thermofisher, South Africa). The cell solution was spun at 800x g for 10 min at room temperature. The supernatant was discarded and cell pellet was re-suspended with 1 mL RPMI-1640 medium (Thermofisher, South Africa) for further cell count and processing.

**RT-PCR**

Skin samples were firstly homogenized in 1ml Qiazol lysis reagent, thereafter RNA was extracted using Qiagen kit according to the manufacturer’s instructions (Qiagen, Germany). First Strand cDNA Synthesis Kit (Roche) was used following the manufacturer’s guidelines to convert RNA into cDNA. The LightCycler® 480 SYBR Green I Master Kit (Roche) was used for this assay. The concentration of mRNA transcripts was normalized to the mRNA level of the beta-actin or *gaphd* housekeeping gene. Relative fold changes in gene expression were calculated using the formula 2^-ΔΔCt^ , a method previously described^2^. Expression levels of each gene were plotted using GraphPad Prism 8 software. The following primers were used.

**Supplementary Table 1:** Primer sequences (Integrated DNA Technologies, USA) used for qRT-PCR

| **Gene name** | **Forward primer sequence (5’ 3’)** | **Reverse primer sequence 5’ 3’)** |
| --- | --- | --- |
| IL-5 | TCA CCG AGC TCT GTT GAC AA | CCA CAC TTC TCT TTT TGG CG |
| IL-13 GEX | CTC CCT CTG ACC CTT AAG GAG | GAA GGG GCC GTG GCG AAA CAG |
| IL-33 | AAC CAG CTG GCT CTA GTG GA | ACT GTG GTG CCT GCT CTT CT |
| IL-25 | CAT TCT TGG CAA TGA TCG TG | GAA GAC CGT CGT GTT GTG GT |
| IL-4 | TCG GCA TTT TGA ACGA GGTC | GAA AAG CCC GAA AGAG TCTC |
| IL-17 | CTC CAG AAG GCC CTCA GACT AC | AGC TTT CCC TCC GCAT TGAC ACAG |
| Beta-actin | TGG AAT CCT GTG GCA TCC AGA AAC | TAA AAC GCA GCT CAG TAA CAG TCC G |

References

1. Jensen, K. B., Driskell, R. R. & Watt, F. M. Assaying proliferation and differentiation capacity of stem cells using  disaggregated adult mouse epidermis. *Nat Protoc* **5**, 898–911 (2010).

2. Livak, K. J. & Schmittgen, T. D. Analysis of relative gene expression data using real-time quantitative PCR and the 2-ΔΔCT method. *Methods* **25**, 402–408 (2001).
